# Safety and efficacy of idarucizumab-assisted thrombolysis in dabigatran-related acute ischemic stroke: a systematic review and single-arm meta-analysis

**DOI:** 10.3389/fneur.2026.1884021

**Published:** 2026-08-20

**Authors:** Xiaoyuan Guo, Hang Xiong, Wenping Li, Qian Liu, Wei Li, Lingbo Kong, Xinxing Lai

**Affiliations:** 1 Department of Neurology, Dongzhimen Hospital, Beijing University of Chinese Medicine, Beijing, China; 2 Department of Neurology, Chifeng Municipal Hospital, Chifeng, China; 3 Acupuncture Department, Huguosi Hospital of Traditional Chinese Medicine Affiliated to Beijing University of Chinese Medicine, Beijing, China; 4 General Practice Department, Gantang Community Health Service Center, Beijing, China; 5 Institute for Brain Disorders, Beijing University of Chinese Medicine, Beijing, China

**Keywords:** acute ischemic stroke, dabigatran, idarucizumab, intravenous thrombolysis, meta-analysis

## Abstract

**Background:**

Intravenous thrombolysis (IVT) in patients with acute ischemic stroke (AIS) related to dabigatran use requires prior anticoagulation reversal. This study aimed to evaluate the safety and efficacy of IVT following idarucizumab-mediated reversal through a systematic single-arm meta-analysis.

**Methods:**

We systematically searched multiple databases. Single-arm studies (≥5 patients) on idarucizumab-facilitated IVT for dabigatran-related AIS were included. Outcome data from DOAC patients without reversal in the Meinel 2023 cohort (*n* = 580) served as a descriptive external reference. Pooled proportions for safety (symptomatic intracranial hemorrhage, sICH) and efficacy (90-day good functional outcome, mRS 0–2) were calculated using a random-effects GLMM.

**Results:**

Eleven single-arm studies (226 patients) were included. The pooled sICH rate was 5.4% (95% CI 2.6–10.6%; *τ*^2^ = 0.362, *Q p* = 0.12). The pooled 90-day good functional outcome rate was 66.6% (95% CI 54.2–77.1%; *τ*^2^ = 0.116). In the external reference cohort, the sICH rate was 3.1% and the good outcome rate was 42.4%. Due to baseline imbalances and lack of confounder adjustment, descriptive comparisons were hypothesis-generating only. The certainty of evidence was rated very low (GRADE).

**Conclusion:**

Current evidence is insufficient to guide routine clinical practice. However, these findings provide useful effect-size estimates (sICH ~5%, good outcome ~67%) for designing future randomised controlled trials. Multicenter prospective registries or pragmatic RCTs are warranted.

## 1 Introduction

### 1.1 Clinical background

Direct oral anticoagulants (DOACs) are widely prescribed for stroke prevention in patients with non-valvular atrial fibrillation and venous thromboembolism (1). Nevertheless, despite optimal anticoagulation, approximately 1–2% of patients still experience an acute ischemic stroke (AIS) annually (2). Intravenous thrombolysis (IVT) remains the cornerstone of reperfusion therapy for these patients. However, current international guidelines consider DOAC intake within 48 h of symptom onset a relative or absolute contraindication to IVT because of concerns about symptomatic intracranial hemorrhage (sICH) (3, 4).

Idarucizumab, a humanized monoclonal antibody fragment, exhibits an affinity for dabigatran approximately 350-fold greater than that of dabigatran for thrombin, which can reverse dabigatran’s anticoagulant activity within minutes (5). It is currently approved for emergency reversal of dabigatran in patients with life-threatening or uncontrolled bleeding, or those requiring urgent surgical or invasive procedures (6).

### 1.2 Evidence gap

Although case reports, case series, and registry studies have suggested the feasibility of administering IVT following idarucizumab reversal (7–10), the existing evidence remains substantially limited. First, most available studies are single-center, retrospective analyses with small sample sizes. Second, outcome definitions and follow-up durations are highly heterogeneous across cohorts. Third, previous systematic reviews have primarily compared overall DOAC-treated patients with non-anticoagulated controls. To date, no study has specifically synthesized single-arm effect estimates for dabigatran-specific reversal prior to thrombolysis, nor provided a structured descriptive reference against non-reversed DOAC cohorts. Available primary studies on idarucizumab-facilitated thrombolysis are limited to small case series (11, 12), and previous systematic reviews have primarily compared IVT outcomes between DOAC users and non-anticoagulated patients rather than addressing this specific reversal strategy.

### 1.3 Study objectives

This study aims to: (1) perform an updated single-arm meta-analysis incorporating the latest evidence up to 2026 to provide pooled event-rate estimates for this strategy, thereby supplying essential effect-size parameters for future research; (2) utilize the largest and most rigorously defined contemporary cohort [Meinel et al. (6)] (3) as a descriptive external reference, presenting its outcomes alongside those of non-reversed DOAC patients to contextualize the observed results; and (3) generate robust effect-size estimates to inform sample size calculation and the design of future confirmatory randomised controlled trials (13).

## 2 Methods

### 2.1 Registration and reporting standards

The study protocol was prospectively registered with PROSPERO (CRD420261378203). The manuscript was prepared and reported in strict accordance with the PRISMA 2020 statement (14) and the MOOSE guidelines (15). Completed checklists are provided in Supplementary material S1.

Following the systematic search, evidence for other DOAC-specific reversal agents (e.g., andexanet alfa) combined with thrombolysis was limited to isolated case reports and fell below the minimum threshold for quantitative synthesis. Furthermore, only one study [Meinel et al. (6)] met the criteria for an external descriptive reference cohort of DOAC-treated patients undergoing thrombolysis without reversal agents while providing complete outcome data. Given these objective constraints in the available evidence and to maintain pharmacological homogeneity, the protocol was amended on the PROSPERO platform on 12 May 2026, after data extraction and quality appraisal but prior to data synthesis. The amendment restricted the analysis to a single-arm meta-analysis of dabigatran reversal with idarucizumab before intravenous thrombolysis (IVT) and designated the Meinel et al. (6) cohort as a descriptive historical reference. The amendment record is publicly accessible (pending editorial review; a screenshot is provided in Supplementary Figure S2). This modification was driven solely by evidence availability and did not introduce outcome-driven selection bias, as it preceded data pooling. However, this design inherently limits comparative inference: conclusions apply strictly to the dabigatran population, and the descriptive reference lacks confounder adjustment and cannot support causal claims.

### 2.2 Search strategy

A comprehensive literature search was conducted from database inception to 29 April 2026, with a supplementary search of ClinicalTrials.gov completed on 4 May 2026. The following electronic databases and registries were queried: Cochrane Library, Embase, Web of Science Core Collection, PubMed, CNKI, VIP, WanFang, SinoMed, and WHO ICTRP. The search strategy was intentionally broad, encompassing all DOAC classes, to ensure that no relevant dabigatran studies were missed due to indexing variations or non-standardised terminology in the literature. After systematic screening, only dabigatran-specific studies met the prespecified inclusion criteria for quantitative synthesis. Using PubMed as an example, the search strategy was constructed as:

(“Direct Oral Anticoagulants”[Mesh] OR dabigatran[tiab] OR rivaroxaban[tiab] OR apixaban[tiab] OR edoxaban[tiab]) AND (“Ischemic Stroke”[Mesh] OR “Ischemic Stroke”[tiab]) AND (“Thrombolytic Therapy”[Mesh] OR “Alteplase”[Mesh] OR “Tenecteplase”[Mesh] OR IVT[tiab]) AND (“Antidotes”[Mesh] OR “Idarucizumab”[Mesh] OR “Andexanet Alfa”[Mesh] OR idarucizumab[tiab])

No study design filters were applied *a priori* to maximise sensitivity. Eligibility screening (restricted to single-arm observational cohorts, case series, and registry reports without mandatory control arms) was performed through independent manual review of titles and abstracts, followed by full-text assessment by two investigators. Complete search strategies for all databases and trial registries are detailed in Supplementary Table S3.

### 2.3 Eligibility criteria

Studies were included if they met the following criteria: (1) adult patients (≥18 years) with confirmed acute ischemic stroke (AIS); (2) prior use of dabigatran (exclusively limited to this DOAC); (3) administration of idarucizumab for reversal before IVT; (4) study designs comprising prospective or retrospective cohorts or case series (≥5 patients). Case reports and smaller case series were excluded to minimize publication bias and to ensure a minimum level of data stability and reliability for safety outcome estimates; and (5) reporting of at least one predefined outcome. Exclusion criteria were: (1) overlapping cohorts or duplicate publications; (2) sample size <5; (3) inability to extract key outcome data; and (4) publications in languages other than English or Chinese.

External historical reference cohort: We selected patients from the Meinel et al. (6) study who received IVT without reversal agents (*n* = 580). This cohort was chosen because it provided a clearly defined external descriptive reference population (DOAC-treated patients undergoing IVT without reversal), a substantial sample size, and no data overlap with the included single-arm studies (6).

Rationale for this selection: Among all systematically identified literature, this remains the only large-scale, multicenter cohort reporting both sICH and 90-day mRS for unreversed DOAC thrombolysis, with explicit stratification between reversed and unreversed groups. Although baseline differences exist between groups (e.g., DOAC composition, stroke severity, mechanical thrombectomy rates; see Table 1; Supplementary Table S4), this reference cohort serves to contextualize the single-arm findings within a real-world framework.

**Table 1:** Baseline characteristics and outcomes of the external descriptive reference cohort [Meinel et al. (6)].

Characteristic	Meinel 2023 cohort
Study design	Retrospective cohort
Country/region	International (64 centers)
Reversal group, *n*	252 (idarucizumab + dabigatran)
Unreversed DOAC group, *n*	580
Dabigatran in unreversed group, *n* (%)	90 (15.5%)
DOAC composition in unreversed group	Mixed: rivaroxaban 258, apixaban 163, edoxaban 68, dabigatran 90, unspecified 1
sICH rate in unreversed group, *n*/*N* (%)	18/580 (3.1)
90-day mRS 0–2 in unreversed group, *n*/*N* (%)	246/580 (42.4)

This cohort is presented exclusively as a descriptive real-world reference. The unreversed DOAC group contains predominantly factor Xa inhibitor users (rivaroxaban, apixaban, edoxaban; 84.3%), with only 90 patients (15.5%) on dabigatran. This composition differs fundamentally from the dabigatran-only meta-analysis population. Therefore, no statistical comparisons were performed between this cohort and the meta-analysis results. All numerical differences should be interpreted as hypothesis-generating only.

### 2.4 Data extraction and outcome definitions

Data extraction was performed independently by two reviewers using a standardised, pilot-tested form. Extracted variables included: first author, publication year, country/region, study design, sample size, age (mean/median), proportion of males, baseline median NIHSS score, DOAC type, time from last dose to thrombolysis, reversal agent dosage, thrombolytic agent and dose, event counts and denominators for all outcomes, and risk of bias assessment details.

Primary outcomes were: (1) symptomatic intracranial hemorrhage (sICH), prioritising definitions from the ECASS-II (16) or NINDS (17) criteria; if unspecified in the original report, the authors’ own definition was retained; and (2) favorable functional outcome at 90 days, defined as a modified Rankin Scale (mRS) score of 0–2 (18).

Secondary outcomes included any intracranial hemorrhage (ICH) and all-cause mortality (90-day mortality was prioritised; in-hospital or discharge mortality was reported separately when available).

Handling of missing data: For the single-arm studies, if a specific outcome was unreported, the study was excluded from the pooled analysis for that outcome without imputation (complete-case approach). For the external historical reference cohort [Meinel et al. (6)], 90-day mortality was not reported by subgroup in the original publication; therefore, this outcome was omitted from the descriptive comparison.

### 2.5 Risk of bias assessment

Given that all included studies were single-arm observational cohorts or case series without concurrent control groups, methodological quality was assessed using the Joanna Briggs Institute (JBI) Critical Appraisal Checklist for Case Series (19). This tool is specifically recommended for evaluating selection, measurement, and attrition biases in single-arm evidence and was pre-specified in the PROSPERO registration (CRD420261378203).

Two reviewers independently evaluated the 11 included single-arm studies. The checklist comprises 10 domains covering inclusion criteria, reliability and validity of condition measurement, consecutive enrolment, completeness of demographic and clinical reporting, clarity of outcome reporting, and appropriateness of statistical methods. Each item was scored as “Yes” (criterion met), “No” (criterion not met), or “Unclear.” Studies with predominantly “Yes” responses were classified as low risk of bias, whereas multiple “No” or critical missing information indicated high risk. Discrepancies were resolved through consensus or consultation with a third senior researcher.

### 2.6 Statistical analysis

The statistical evaluation comprised two distinct components: a single-arm meta-analysis of proportions and a descriptive reference to an external historical cohort. All analyses and reporting adhered to the PRISMA 2020 statement and the Synthesis Without Meta-analysis (SWiM) 2020 guidelines.

#### 2.6.1 Single-arm meta-analysis

Pooled proportions for binary outcomes were estimated using a random-effects generalized linear mixed model (GLMM) with a logit link, which handles sparse data (including zero-event studies) without continuity corrections (20).

Each study contributed the number of events and the corresponding total sample size. Between-study variance (*τ*^2^) was estimated using restricted maximum likelihood (REML). The model solved for the pooled proportion and its standard error directly from the binomial likelihood, without requiring a pre-specified weighting matrix. Pooled proportions were back-transformed via the inverse logit function to yield clinically interpretable proportions with 95% confidence intervals (CIs).

Heterogeneity was quantified using *τ*^2^, the 95% prediction interval, and Cochran’s *Q* test (21, 22). When substantial heterogeneity was present (*Q* test *p* < 0.10), pre-specified subgroup analyses and leave-one-out sensitivity analyses were performed.

All computations were performed in R software (version 4.5.3) using the meta package (23). The metaprop function (specified with method = “GLMM”) was employed for primary pooling. Results were visualized using forest plots. Complete analytical code, raw data matrices, and model diagnostic outputs are provided in Supplementary materials S5–S7.

#### 2.6.2 Descriptive analysis of the external historical cohort

For the external historical reference cohort, raw event counts and proportions were extracted from Meinel et al. (6) for the idarucizumab-reversal group and the unreversed DOAC group (6). Given the observational nature of this descriptive juxtaposition and the absence of propensity score matching or rigorous covariate adjustment, no effect measures (e.g., OR, RR) were calculated, and no formal statistical inference was performed. This descriptive approach aligns with SWiM guidelines for non-synthesized evidence (24) and serves solely to contextualize the single-arm findings within a real-world clinical background.

#### 2.6.3 Subgroup, sensitivity analyses, and publication bias assessment

Subgroup Analyses: For key single-arm outcomes (sICH rate and 90-day favorable functional outcome), analyses were stratified by outcome assessment timing (in-hospital vs. 90 days) and thrombolytic agent (alteplase vs. tenecteplase). Subgroup differences were formally evaluated using interaction tests.

Sensitivity Analyses: Robustness was assessed through leave-one-out sequential exclusion. Additionally, analyses were repeated after excluding studies with very small sample sizes (<10 patients). To address potential heterogeneity in sICH definitions, a pre-specified sensitivity analysis was conducted: studies not employing internationally recognized criteria (ECASS-II (16) or SITS-MOST (17)) were sequentially excluded. If the pooled sICH proportion varied by <1.5 percentage points, definitional differences were deemed unlikely to alter the primary conclusion. Full subgroup and sensitivity analysis results, including stratification by sICH definition, are detailed in Supplementary Table S8.

Publication Bias: When at least 10 studies were available, publication bias was assessed using Doi plots with the LFK index (25). Detailed methodology is provided in Supplementary material S5.

Protocol Registration: The study protocol was prospectively registered in PROSPERO (CRD420261378203).

## 3 Results

### 3.1 Study selection

**Figure 1:**
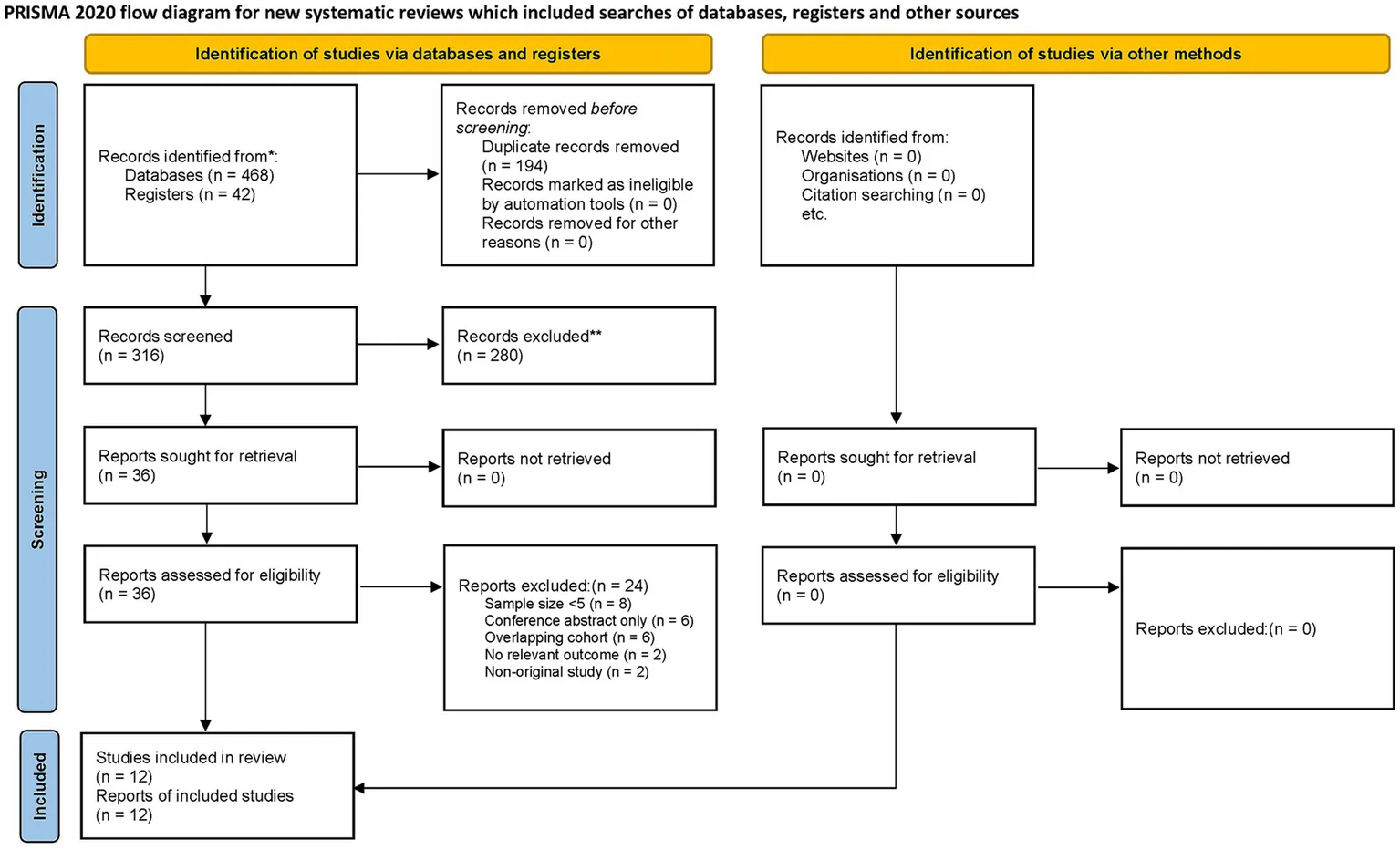
PRISMA 2020 flow diagram of study selection. Records identified from databases and clinical trial registers were deduplicated. After title/abstract screening, full-text articles were assessed for eligibility. Studies were excluded for prespecified reasons including small sample size, conference-only publication, overlapping cohorts, or lack of relevant outcomes. Twelve studies were ultimately included: 11 for single-arm meta-analysis and 1 as an external historical reference cohort for descriptive comparison.

A total of 510 records were identified through database searches (*n* = 468) and clinical trial registries (*n* = 42). An additional 31 records were retrieved from ClinicalTrials.gov. After duplicate removal, 316 unique records remained. Following title and abstract screening, 280 records were excluded. Full-text assessment of the remaining 36 articles led to the exclusion of 24 studies. Primary reasons for exclusion were: insufficient sample size (<5 patients; *n* = 8), conference abstracts (*n* = 6), overlapping cohorts (*n* = 6), absence of predefined outcomes (*n* = 2), and non-primary research designs (*n* = 2). Ultimately, 11 single-arm studies (5, 8, 10–13, 25–29) and one external historical reference cohort [Meinel et al. (6)] met the eligibility criteria. The detailed selection process is illustrated in the PRISMA flow diagram (Figure 1).

### 3.2 Study characteristics

#### 3.2.1 Single-arm studies

Eleven independent studies comprising 226 patients were included (5, 8, 10–13, 25–29). Publication years ranged from 2017 to 2025. Ten studies were retrospective cohorts, and one was prospective [Frol et al. (10)]. Geographically, studies originated from Europe (Germany, Italy, Poland, Latvia, Greece, Slovenia, Czech Republic, Austria) and the Asia-Pacific region (New Zealand, Australia, Taiwan, China). All patients were diagnosed with dabigatran-associated acute ischemic stroke (AIS) and received a standard 5 g dose of idarucizumab for reversal prior to intravenous thrombolysis (IVT). Ten studies used alteplase, while one employed tenecteplase. Definitions of symptomatic intracranial hemorrhage (sICH) varied across studies (ECASS-II, SITS-MOST, or author-defined); the impact of this heterogeneity was evaluated in sensitivity analyses (Section 3.6). Baseline characteristics and outcome data for all included studies are summarized in Table 2, and pooled baseline characteristics are presented in Table 3.

**Table 2:** Baseline characteristics of the 11 included single-arm studies on thrombolysis after idarucizumab reversal.

Study (year)	Country	Study design	Sample size	Age, years	Male (%)	Baseline NIHSS	Thrombolytic agent	sICH (*n*/*N*)	Any ICH (*n*/*N*)	Mortality (*n*/*N*)	Good functional outcome (mRS 0–2) (*n*/*N*)	Outcome assessment time
Wlodarczyk 2023 (11)	Poland	Retrospective cohort	19	74 (IQR 68–81)	68.4	8	Alteplase	2/19	3/19	1/19	13/19	Discharge
Kikule 2022 (26)	Latvia	Retrospective cohort	9	75.7 (mean)	77.8	9	Alteplase	1/9	1/9	1/9	4/9	Discharge
Kermer 2020 (13)	Germany	Retrospective cohort	80	77	63.7	9 (1–42)	Alteplase	0/80	0/80	3/80	NR	Not available
Beharry 2020 (12)	Australia/New Zealand	Retrospective cohort	13	79 (IQR 69–85)	69.2	6 (4–21)	Tenecteplase	0/13	2/13	2/13	8/13	90 days
Fang 2019 (27)	Taiwan, China	Retrospective cohort	10	71.1 (mean)	60.0	16	Alteplase	1/10	3/10	1/10	NR	Not available
Sanák 2018 (25)	Czech Republic	Retrospective cohort	13	70 (mean)	53.8	7	Alteplase	1/13	2/13	3/13	10/13	90 days
Pikija 2017 (29)	Austria	Retrospective cohort	5	75	60.0	10	Alteplase	0/5	0/5	0/5	NR	Not available
Tse 2017 (28)	New Zealand	Retrospective cohort	6	68	50.0	21	Alteplase	1/6	1/6	1/6	2/6	90 days
Frol 2021 (10)	Slovenia	Prospective cohort	22	75	50.0	10.5	Alteplase	1/22	4/22	3/22	19/22	90 days
Theodorou 2024 (5)	Greece	Retrospective cohort	10	73.7	50.0	12	Alteplase	1/10	2/10	2/10	5/10	90 days
Romoli 2023 (8)	Italy	Retrospective cohort (matched)	39	75.3	46.2	10	Alteplase	4/39	9/39	7/39	25/39	90 days

IQR, interquartile range; mRS, modified Rankin Scale; NIHSS, National Institutes of Health Stroke Scale; NR, not reported; sICH, symptomatic intracranial hemorrhage. All patients received idarucizumab (5 g) prior to thrombolysis. Good functional outcome was defined as mRS 0–2. For studies reporting both discharge and 90-day outcomes, 90-day data were prioritized for the main analysis; discharge data are presented descriptively. Mortality and functional outcomes reflect the longest follow-up reported per study. Age and baseline NIHSS are reported as median (IQR), mean, or range according to original publications without data transformation. Outcomes are expressed as events/total patients (*n*/*N*).

**Table 3:** Summary of baseline characteristics of the 11 included single-arm studies.

Characteristic	Pooled estimate
Number of studies	11
Total patients	226
Age, median (range), years	74 (68–79)
Male, %	58.2
Baseline NIHSS, median (IQR)	10 (8–12)
Thrombolytic agent: Alteplase, *n* (%)	10 (90.9)
Thrombolytic agent: Tenecteplase, *n* (%)	1 (9.1)
sICH definition: ECASS-II/III, *n* (%)	7 (63.6)
sICH definition: SITS-MOST, *n* (%)	2 (18.2)
sICH definition: Author-defined, *n* (%)	2 (18.2)

Data are presented as pooled medians (ranges) or proportions across studies. Individual study characteristics are detailed in Table 2.

#### 3.2.2 External historical reference cohort

The external reference cohort was derived from Meinel et al. (6), comprising 580 DOAC-treated patients who underwent IVT without reversal. Of these, only 90 (15.5%) were taking dabigatran; the remainder were on factor Xa inhibitors (rivaroxaban 258, apixaban 163, edoxaban 68). This cohort is used exclusively as a descriptive contextual reference, not as a control arm or for direct comparative inference, due to fundamental differences in DOAC composition and baseline characteristics relative to the dabigatran-only meta-analysis population. Detailed baseline data are presented in Table 1.

### 3.3 Risk of bias assessment

**Figure 2:**
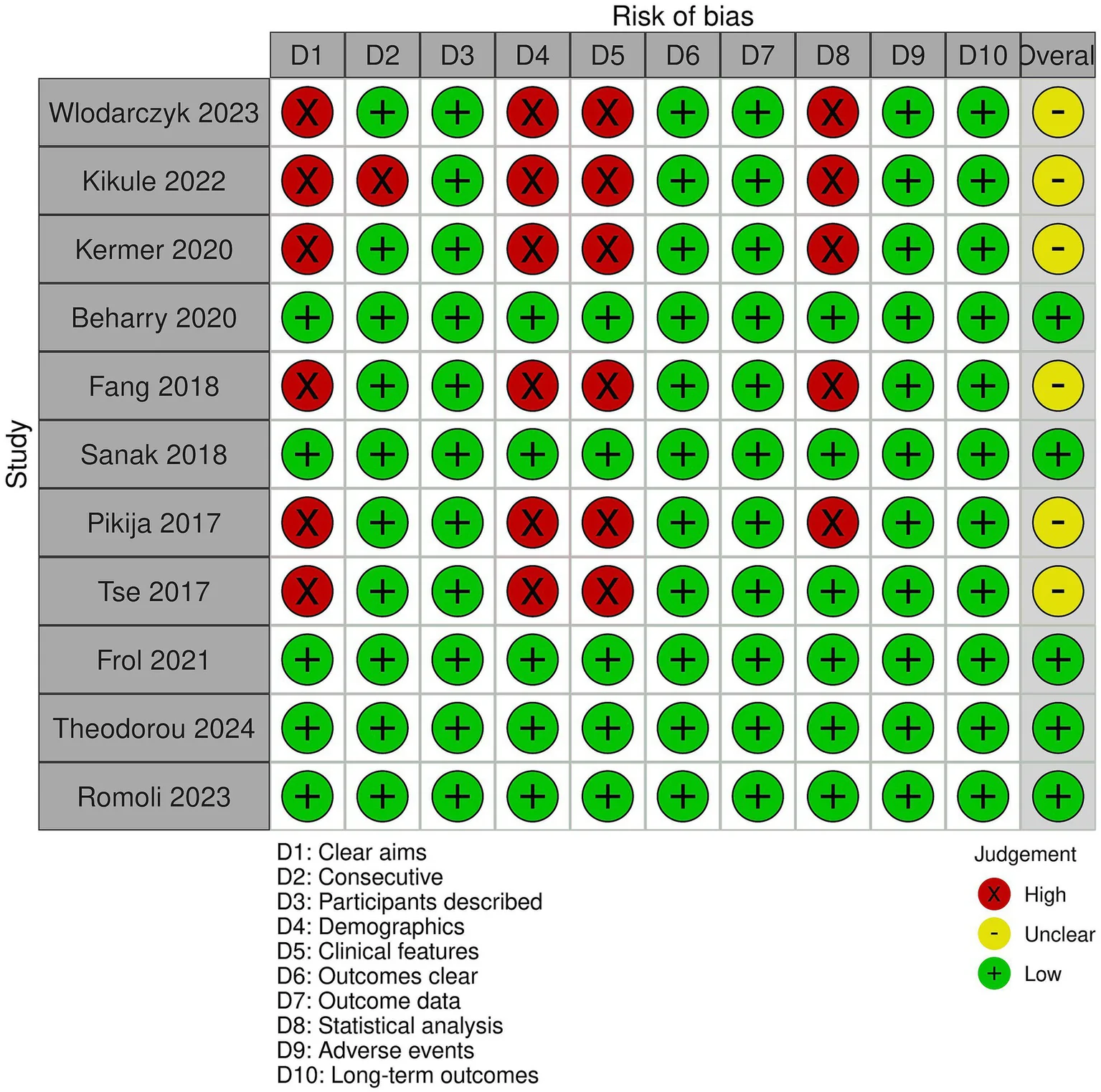
Risk of bias assessment using the JBI Critical Appraisal Checklist for Case Series. Each row represents an included study. Green circles (+) indicate low risk (Yes), yellow circles (−) indicate unclear risk, and red circles (×) indicate high risk (No). Overall judgment was determined by the predominant domain rating; studies with ≥3 high/unclear domains were classified as unclear risk. D1–D10 correspond to: D1 Clear aims, D2 consecutive enrollment, D3 participants clearly described, D4 demographics reported, D5 clinical characteristics reported, D6 outcomes/follow-up clearly reported, D7 outcome data provided, D8 statistical analysis included, D9 adverse events reported, D10 long-term outcomes reported. Detailed domain judgments and full checklist questions are provided in Supplementary material S9.

Methodological quality was independently evaluated using the JBI Critical Appraisal Checklist for Case Series (19). Discrepancies were resolved through discussion or consultation with a third reviewer. Assessments revealed variability in risk of bias across domains. High risk was predominantly observed in consecutive enrollment (Item 2) and complete inclusion of eligible participants (Item 3), particularly in retrospective single-center studies that lacked explicit enrollment protocols or exhibited selective reporting. Conversely, multicenter registries or studies with clearly documented consecutive enrollment [e.g., Beharry et al. (12), Sanák et al. (25), Frol et al. (10), Theodorou et al. (5), Romoli et al. (8)] were rated low risk. Regarding outcome reporting (Item 6), several studies [Wlodarczyk et al. (11), Kikule et al. (26), Kermer et al. (13), Fang et al. (27), Pikija et al. (29)] only reported in-hospital outcomes without 90-day functional follow-up, resulting in high risk for long-term endpoints; studies providing complete 90-day data were rated low risk. All studies scored low risk for clarity of objectives, participant description, demographic/clinical reporting, and statistical appropriateness. Overall, the risk of bias was primarily driven by enrollment continuity and completeness of outcome reporting. However, sensitivity analyses excluding studies with high-risk domains yielded sICH estimates consistent with the primary analysis (range 5.1–6.2%), suggesting that the core safety estimate is relatively robust to methodological heterogeneity. Domain-level assessments are detailed in Figure 2.

### 3.4 Single-arm meta-analysis results

#### 3.4.1 Symptomatic intracranial hemorrhage (sICH)

**Figure 3:**
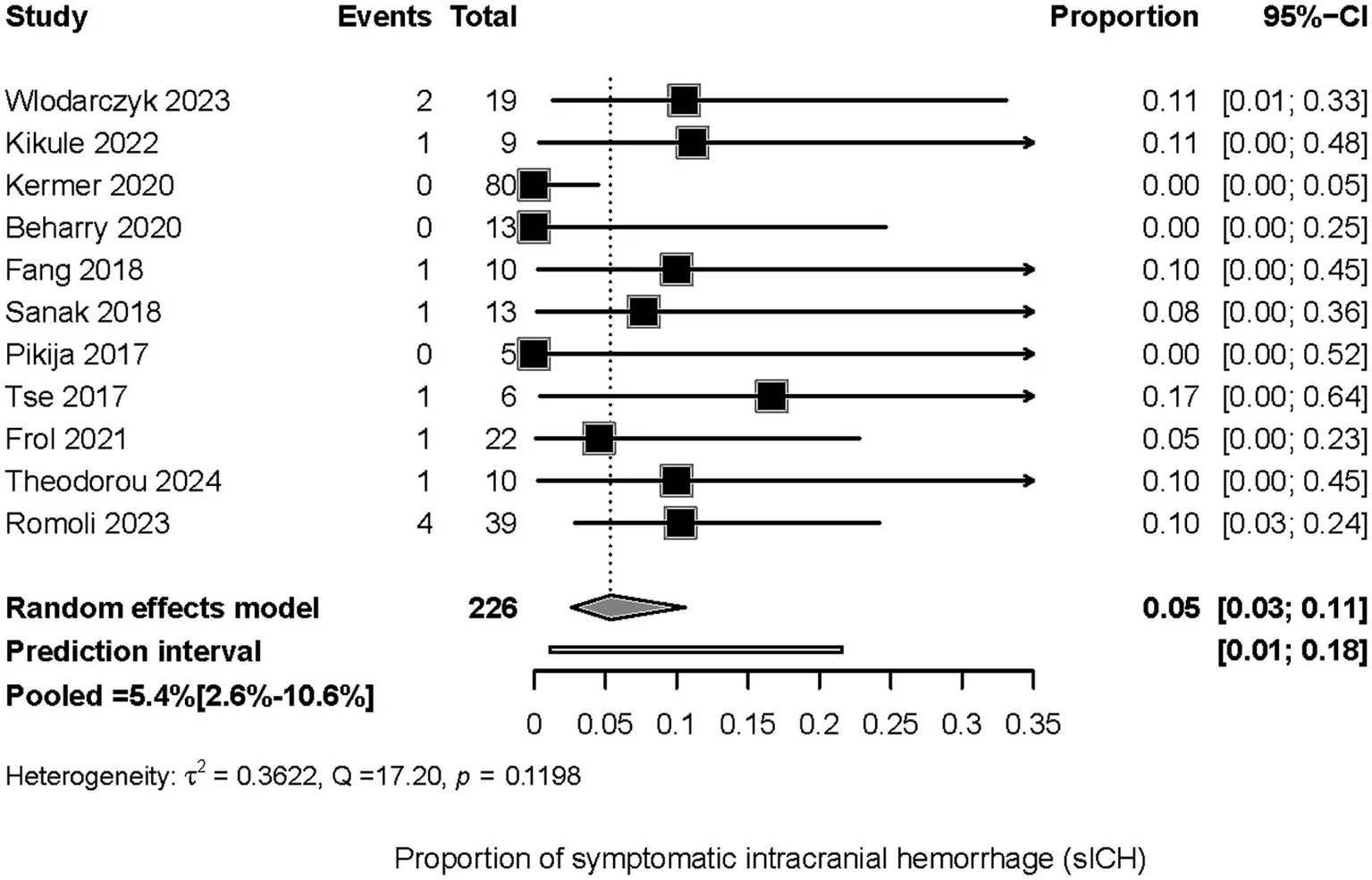
Forest plot summarizing the pooled proportion of symptomatic intracranial hemorrhage (sICH) across 11 single-arm studies. Individual study estimates are represented by black squares (size proportional to the inverse-variance weight), with horizontal lines denoting 95% confidence intervals (CIs). The vertical dotted line indicates the null value (0%). The gray diamond with a black border at the bottom represents the pooled estimate derived from a random-effects generalized linear mixed model (GLMM) with a logit link, along with its 95% CI. The pooled incidence of sICH was 5.4% (95% CI: 2.6–10.6%). Between-study heterogeneity was estimated at *τ*^2^ = 0.362 (Cochran’s *Q* = 17.20, df = 10, *p* = 0.120). The 95% prediction interval (PI) was 1.4–18.5%, indicating the expected range of true sICH rates in future clinical settings with similar patient characteristics and treatment protocols. Studies reporting zero events are appropriately accounted for within the GLMM framework without requiring continuity corrections. SICH, symptomatic intracranial hemorrhage; CI, confidence interval; PI, prediction interval; GLMM, generalized linear mixed model.

All 11 studies reported sICH events, with 12 cases identified among 226 patients. Random-effects generalized linear mixed model (GLMM) pooling yielded a sICH rate of 5.4% (95% CI 2.6–10.6%), with between-study variance *τ*^2^ = 0.362 (Cochran’s *Q* test, *p* = 0.120). The 95% prediction interval (PI) ranged from 1.4 to 18.5%, indicating the plausible range of true sICH rates in future similar studies (Figure 3). Subgroup analyses stratified by outcome assessment timing (in-hospital vs. 90-day) and thrombolytic agent (alteplase vs. tenecteplase) revealed no statistically significant differences (all *p* for interaction > 0.05).

Notably, the pooled sICH estimate (5.4%) was derived from studies with available neuroimaging follow-up (overall follow-up rate: 81.6%). In the external historical reference cohort, 47 patients (18.4% of the reversal group) lacked follow-up imaging, with unreported reasons for loss, constituting non-random missing data, which poses a potential detection bias given that sICH is the primary safety endpoint, this attrition introduces potential detection bias, and the true incidence may be systematically under- or overestimated. Consequently, the current sICH estimate should be interpreted as a conditional reference.

To evaluate the impact of varying sICH definitions, a pre-specified sensitivity analysis stratified studies by diagnostic criteria: seven studies using ECASS-II/III criteria yielded a pooled rate of 0.9% (95% CI 0.0–6.7%), while two studies using SITS-MOST criteria yielded 6.1% (95% CI 0.8–34.6%). The between-group difference was not statistically significant (*p* for interaction = 0.162).

#### 3.4.2 90-day favorable functional outcome (mRS 0–2)

**Figure 4:**
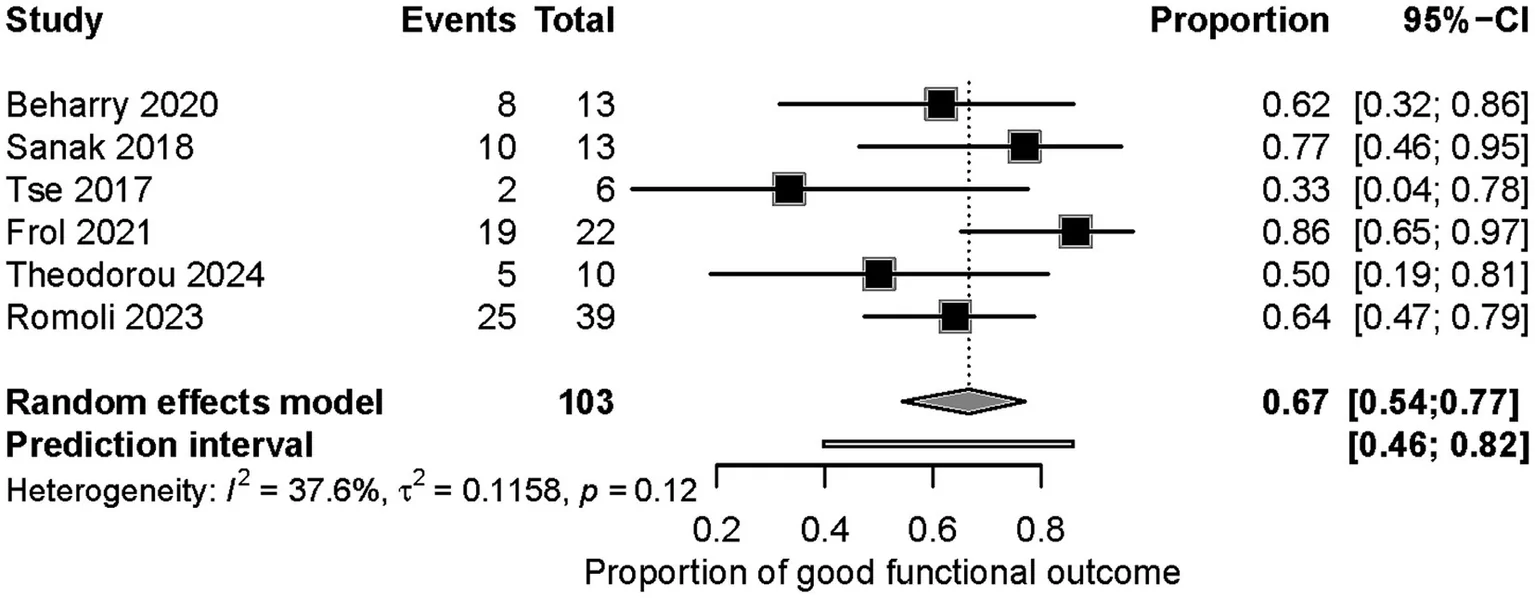
Forest plot of the pooled proportion of good functional outcome (modified Rankin Scale score 0–2 at 90 days) across the six single-arm studies. Proportions were pooled using a random-effects generalized linear mixed model (GLMM) with a logit link. The size of each square is proportional to the study weight. Horizontal lines represent 95% confidence intervals (CIs). The gray diamond with a black border indicates the pooled proportion with its 95% CI. Between-study variance was *τ*^2^ = 0.116 (95% CI 0.000–0.542). The 95% prediction interval (PI) for the true good functional outcome rate in a future setting with similar patient characteristics and treatment conditions was 46.1–82.3%.

Six studies reported 90-day functional outcomes, encompassing 103 patients, of whom 69 achieved an mRS score of 0–2. The pooled favorable outcome rate was 66.6% (95% CI 54.2–77.1%). Moderate heterogeneity was observed (*τ*^2^ = 0.116, 95% CI 0.000–0.542), with a 95% PI of 46.1–82.3%, reflecting the expected variability across future clinical settings (Figure 4). Five additional studies reported only discharge functional status (range 44–68%) and were excluded from the primary 90-day analysis due to incomplete follow-up.

### 3.5 Other outcomes

**Figure 5:**
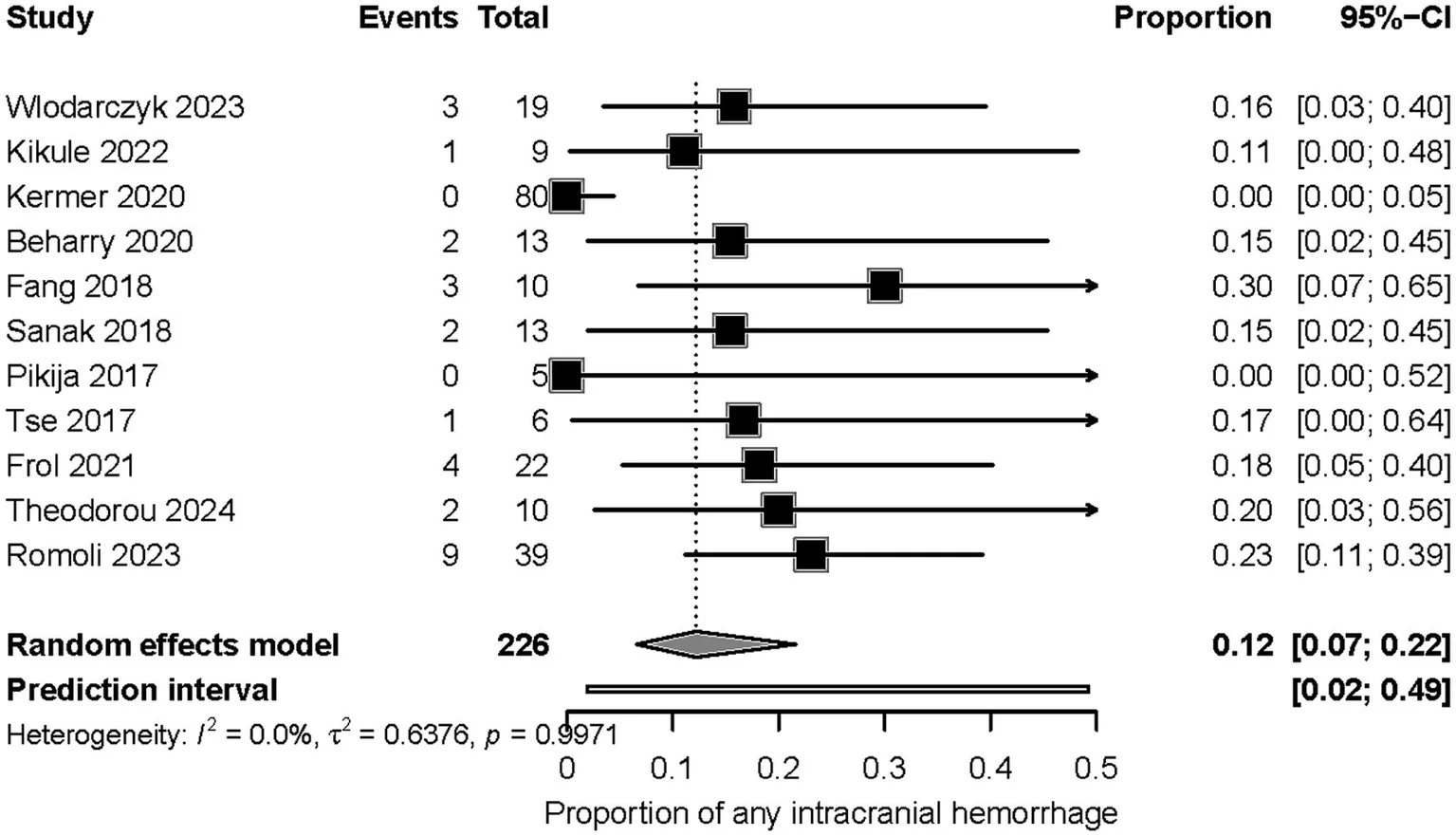
Forest plot of the pooled proportion of any intracranial hemorrhage (any ICH) across the 11 single-arm studies. Proportions were pooled using a random-effects generalized linear mixed model (GLMM) with a logit link. The size of each square is proportional to the study weight. Horizontal lines represent 95% confidence intervals (CIs). The gray diamond with a black border indicates the pooled proportion with its 95% CI. Heterogeneity was low. Most hemorrhagic transformations were asymptomatic; only one event was symptomatic (already counted in sICH).

All 11 studies reported any ICH events, with 27 cases identified. The pooled incidence was 12.2% (95% CI 6.6%--21.6%) (Figure 5). The vast majority represented asymptomatic hemorrhagic transformation; only one event was symptomatic and was accordingly captured in the sICH analysis.

In-hospital mortality was reported in four studies [Kikule et al. (26), Kermer et al. (13), Fang et al. (27), Pikija et al. (29)], with 5 deaths among 104 patients, yielding a pooled rate of 4.8% (95% CI 2.0%–11.0%). Ninety-day.

mortality was reported in five studies [Beharry et al. (12), Sanák et al. (25), Frol et al. et al. (10), Romoli et al. (8), Tse et al. (28)], with 16 deaths among 93 patients, corresponding to a pooled rate of 17.2% (95% CI 10.8%--26.3%). These subsets were non-overlapping; therefore, no statistical interaction test was performed. The corresponding forest plot is provided in Supplementary Figure S10.

### 3.6 Subgroup and sensitivity analyses

Subgroup analysis by thrombolytic agent: Stratification by alteplase (10 studies) versus tenecteplase (1 study) showed no statistically significant difference in pooled sICH proportions (*p* for interaction = 0.205; Supplementary Figure S12).

Leave-one-out sensitivity analysis: Sequential exclusion of individual studies yielded pooled sICH proportions ranging from 4.6 to 8.2%, all within the 95% CI of the primary estimate (5.4%). Exclusion of the smallest study [Pikija et al. (29)] gave a pooled proportion of 5.8% (Supplementary Figure S13).

Sensitivity analysis for sICH definition: To further assess the robustness of the primary sICH estimate, we sequentially excluded studies that did not employ internationally recognized criteria (ECASS-II or SITS-MOST). Nine studies explicitly used ECASS-II or SITS-MOST criteria; two studies [Kermer et al. (13), Pikija et al. (29)] reported only “radiographic hemorrhage” without explicit symptomatic criteria (13, 29). Excluding these two studies (remaining *n* = 210) yielded a pooled sICH of 5.1% (95% CI 2.4–10.4%). Further excluding Kermer et al. (13) (*n* = 80) left eight studies (*n* = 130), yielding a pooled proportion of 6.2% (95% CI 2.9–12.8%). Detailed data are provided in Supplementary Table S8.

### 3.7 Descriptive reference

In the Meinel cohort, the unreversed DOAC subgroup (*n* = 580) had a raw sICH rate of 3.1% (18/580) and a 90-day mRS 0–2 rate of 42.4% (246/580) [Meinel et al. (6)]. Baseline characteristics of this cohort differed from the meta-analysis population (Table 1); therefore, these data are presented for descriptive contextualization only.

### 3.8 Publication Bias assessment

**Figure 6:**
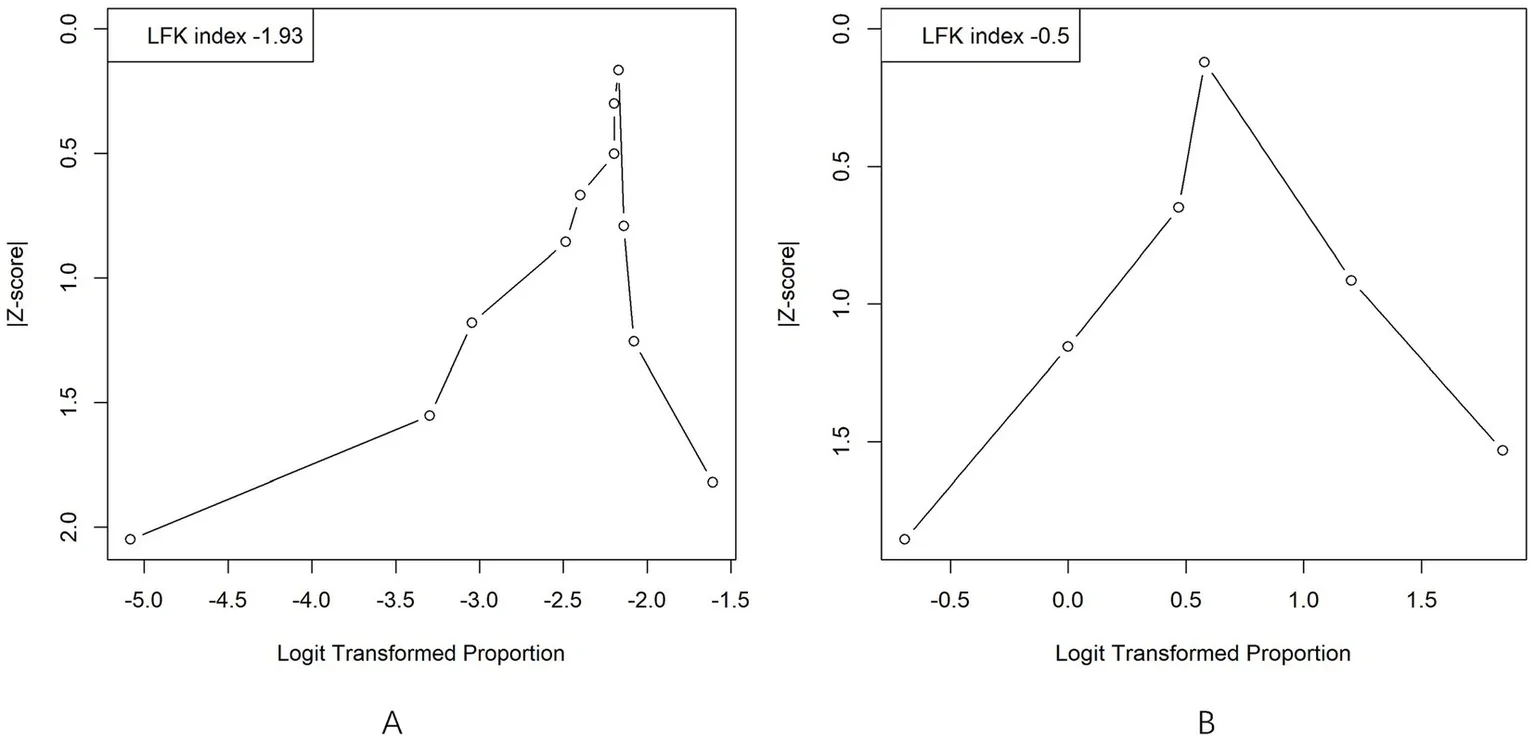
Doi plots for publication bias assessment. **(A)** Symptomatic intracranial hemorrhage (sICH). The LFK index was −1.93 (|LFK| between 1 and 2 indicates moderate asymmetry). **(B)** 90-day good functional outcome (mRS 0–2). The LFK index was −0.5 (|LFK| < 1 indicates minor or no asymmetry). Given the limited number of studies (*k* = 6 for the 90-day outcome), these results are exploratory. The performance of publication bias assessment in single-arm proportion meta-analyses is limited; therefore, these findings should be interpreted with caution and in conjunction with other sources of heterogeneity.

Publication bias was assessed using the Doi plot and LFK index (Figure 6). For the primary safety outcome (sICH), the LFK index was −1.93, indicating moderate asymmetry, suggesting potential small-study effects or heterogeneity due to the limited number of studies (*k* = 11). In contrast, the LFK index for 90-day functional outcome was −0.5, suggesting no significant asymmetry, although this finding is based on only six studies and should be interpreted with particular caution (25).

Asymmetry in Doi plots may reflect clinical or methodological heterogeneity rather than publication bias alone. The statistical power to detect small-study effects in single-arm proportion meta-analyses is inherently limited, rendering these findings exploratory. Furthermore, the small number of included studies (*k* = 11, with only 6 studies contributing to the 90-day functional outcome analysis) limits the reliability of formal publication bias assessment. Therefore, we cannot rule out the possibility of selective reporting, and these results should be interpreted with caution.

## 4 Discussion

### 4.1 Principal findings

This systematic synthesis offers preliminary observations suggesting that idarucizumab-facilitated intravenous thrombolysis (IVT) may represent a feasible strategy for patients experiencing acute ischemic stroke (AIS) while under dabigatran anticoagulation. The core finding of this analysis is the potential establishment of a “safety net” conferred by specific reversal. By rapidly restoring hemostatic competence, idarucizumab may help address the primary safety concern associated with thrombolysis in anticoagulated patients—namely, the risk of symptomatic intracranial hemorrhage (sICH). Our analysis suggests that this strategy converts a formally contraindicated scenario into a clinically manageable one, with a hemorrhagic risk profile that appears tolerable within the context of this high-risk population.

Concurrently, the observed efficacy signals suggest potential benefit. The high rate of functional independence suggests that the restoration of coagulation balance does not necessarily come at the cost of therapeutic efficacy. Instead, this approach may enable a subset of patients to derive the established benefits of reperfusion therapy. Furthermore, the temporal analysis of mortality suggests that late deaths predominate, which may imply that the adverse outcomes in this cohort are driven more by the severity of the underlying cerebrovascular event and comorbidities rather than by the pharmacological intervention itself.

### 4.2 Contextualisation with prior literature

When contextualized against the broader literature, our findings offer a nuanced perspective on the risk–benefit trade-off of reversal strategies. Specifically, we contextualize our findings alongside the Meinel et al. (6) cohort, which represents the largest real-world reference for unreversed DOAC-treated patients undergoing IVT.

A critical observation is the disparity in functional outcomes between our reversal cohort and the Meinel reference. While the Meinel cohort reported a 90-day favorable outcome rate of approximately 42%, our pooled estimate was markedly higher. We hypothesize that this difference may reflect potential clinical benefit: idarucizumab administration likely selects for a population where timely reperfusion is prioritized, whereas the absence of reversal may lead to treatment delays or suboptimal dosing in clinical practice. This raises the possibility that the benefit of reversal may extend beyond mere safety, potentially enabling effective treatment delivery.

Regarding safety, the sICH rate in our analysis was numerically higher than the 3.1% observed in the Meinel unreversed cohort but falls within the range of historical benchmarks for non-anticoagulated patients (typically ranging from 2 to 7%) (16, 17). This finding is hypothesis-generating; it offers a preliminary suggestion that idarucizumab may not amplify bleeding risk compared to the general stroke population, despite the patients’ prior anticoagulation. This observation warrants further investigation into whether anticoagulated patients are inherently at “excess risk” for sICH post-thrombolysis if properly reversed.

### 4.3 Clinical implications and future trial design

Idarucizumab reverses dabigatran’s anticoagulant effect within minutes by binding with 350-fold higher affinity than dabigatran for thrombin, effectively neutralizing its activity and restoring coagulation competence. This rapid reversal transforms patients who would otherwise be ineligible for IVT-due to guideline contraindications-into candidates for reperfusion therapy. Our findings raise the possibility that this mechanistic advantage could be associated with clinically meaningful outcomes, with approximately two-thirds of treated patients achieving functional independence. While the upfront cost of idarucizumab is substantial, the observed functional benefit—potentially reducing long-term disability and dependency—raises the possibility that this expenditure could be offset by downstream savings in chronic care. Dedicated cost-effectiveness analyses are warranted to inform reimbursement decisions. Future studies should evaluate the cost-effectiveness of this strategy to inform reimbursement decisions and guideline recommendations. Our findings provide exploratory quantitative reference points for this decision-making framework, but must be interpreted strictly within a hypothesis-generating context.

As a single-arm synthesis without concurrent controls or confounding adjustment, this analysis cannot substitute for randomised controlled trials (RCTs) or inform routine clinical guidelines. However, the derived absolute event rates (sICH ~5.4%; 90-day mRS 0 to 2 ~66.6%) offer useful *a priori* parameters for future trial design. For instance, assuming a 5% sICH risk and a 67% functional independence rate as baseline expectations, investigators can more accurately calculate sample sizes, optimise inclusion criteria, and reduce trial costs. These estimates require definitive validation through adequately powered, prospectively designed RCTs before translation into practice. We acknowledge that a formal cost-effectiveness analysis was beyond the scope of this study; therefore, our remarks on cost are speculative and should be interpreted as hypothesis-generating.

### 4.4 Limitations

Several limitations warrant consideration.

1. Single-arm synthesis: All included studies were observational (retrospective cohorts or case series), introducing potential selection bias (e.g., preferential reversal in lower-severity patients). Heterogeneity in sICH definitions (ECASS-II, SITS-MOST, NINDS, or institutional criteria) was addressed via definition-driven sensitivity analysis. Excluding two studies lacking standardised criteria [Kermer et al. (13), Pikija et al. (29)] yielded a pooled sICH rate of 5.1% (vs. 5.4% primary), and further exclusion of the largest ambiguous cohort [Kermer et al. (13), *n* = 80] yielded 6.2%. These minimal deviations suggest that definitional variability did not materially alter the core safety conclusion. Additionally, incomplete 90-day follow-up in several studies may overestimate functional recovery, and small sample sizes (<10 patients) in some reports could influence pooled estimates, though leave-one-out analyses demonstrated robustness.
2. Descriptive reference limitations: The Meinel et al. (6) cohort serves exclusively as an observational benchmark. Baseline differences in DOAC type, NIHSS, and thrombectomy rates, coupled with the absence of propensity matching or multivariable adjustment, preclude causal attribution. Notably, 47/252 patients (18.4%) in the reversal subgroup lacked follow-up neuroimaging for any ICH assessment, with unreported reasons for attrition. This non-random missingness introduces substantial detection or attrition bias, as imaging completion may correlate with clinical trajectory (e.g., early death/discharge or mild symptoms precluding routine scans). Without individual patient data, worst−/best-case imputation was infeasible, and the certainty of evidence for any ICH is accordingly downgraded. Furthermore, 90-day mortality was not reported in the reference cohort, limiting long-term safety contextualisation.
3. Additional constraints: Potential language bias (English/Chinese restriction), exclusion of unpublished data, and inability to stratify by thrombolysis time window or baseline coagulation parameters due to sparse primary reporting. The analysis is strictly confined to dabigatran; evidence for other DOACs and their respective reversal agents remains insufficient for quantitative synthesis.

The external Meinel cohort, while providing a valuable real-world reference, has limited comparability with our meta-analysis population. Only 15.5% of the unreversed group were taking dabigatran; the majority were on factor Xa inhibitors with different pharmacokinetic profiles and bleeding risks. This compositional heterogeneity, combined with unadjusted baseline differences, precludes any causal or comparative interpretation. We emphasize that all references to this cohort in our manuscript are strictly descriptive and hypothesis-generating.

Definitional heterogeneity in sICH (ECASS-II vs. SITS-MOST) was addressed through sensitivity analyses. Excluding studies without standardised criteria yielded pooled estimates (5.1–6.2%) that remained within the 95% CI of the primary analysis (5.4%), indicating that variability in sICH definitions did not materially affect the core safety conclusion.

The moderate asymmetry observed in the sICH analysis (LFK = −1.93) suggests potential small-study effects or heterogeneity, likely due to the limited number of included studies (*k* = 11) and the inclusion of several small case series. Notably, the leave-one-out sensitivity analysis yielded pooled sICH estimates ranging from 4.6 to 8.2%, all within the 95% CI of the primary estimate, indicating that no single study disproportionately influenced the pooled result. Therefore, while the LFK index suggests possible small-study effects, the core safety estimate remains relatively robust. Nonetheless, the pooled sICH estimate should be interpreted with caution, and this finding underscores the need for larger, prospective studies to confirm the safety profile. We acknowledge that the high acquisition cost of idarucizumab is a significant barrier to clinical implementation. However, as this study did not perform a formal cost-effectiveness analysis, any discussion of cost implications remains speculative and should be interpreted with caution. The observed discrepancy between ECASS-II/III (0.9%) and SITS-MOST (6.1%) subgroups may reflect differences in study design, patient selection criteria, or threshold definitions for symptomatic hemorrhage. SITS-MOST requires only a ≥ 2-point NIHSS increase, whereas ECASS-II requires a ≥ 4-point increase. This definitional difference is well recognized and may contribute to the numerical variation, though the interaction test was not statistically significant. These findings highlight the heterogeneity inherent in pooling data across different sICH definitions and underscore the need for standardised criteria in future studies.

### 4.5 Future directions

Future research should prioritise well-designed, multicentre prospective registries or pragmatic RCTs with standardised eligibility criteria, uniform outcome definitions, and mandatory 90-day follow-up. Key unanswered questions include the interaction between DOAC class, reversal agent, thrombolytic type, and treatment window, as well as the comparative effectiveness and cost-utility of idarucizumab versus andexanet alfa for factor Xa inhibitors (30). Stratified analyses by stroke severity and biomarker-guided reversal thresholds will further refine personalised decision-making. Accumulating evidence from observational syntheses, including the present study, may inform future discussions regarding stroke management guidelines for reversal-guided thrombolysis in dabigatran-treated patients, particularly in centers with established protocols for rapid idarucizumab administration.

### 4.6 Strengths

Despite these limitations, this synthesis offers several methodological advantages: (i) it represents one of the most comprehensive evidence integrations in this niche, updated through 2026; (ii) strict pharmacological homogeneity by focusing exclusively on dabigatran, thereby mitigating inter-DOAC variability; (iii) transparent stratification of in-hospital vs. 90-day mortality to underscore prognostic time-dependency; (iv) explicit incorporation of a descriptive historical benchmark to address the core clinical reversal dilemma; and (v) rigorous sensitivity and subgroup analyses confirming estimate stability.

### 4.7 Certainty of evidence (GRADE assessment)

Following GRADE methodology (31), we rated the certainty of evidence for primary outcomes in an exploratory manner. While GRADE is traditionally designed for comparative effect estimates, its application to single-arm proportions provides transparent benchmarking for evidence interpretation. The starting certainty was set at “Low” (observational single-arm design). This was downgraded by one level for indirectness (single-arm synthesis with a descriptive historical reference cannot address comparative effectiveness) and by one level for imprecision (wide confidence intervals and limited sample size). Consequently, both sICH and 90-day functional outcome were rated as Very Low certainty. This rating indicates that the true effect may substantially differ from the estimate and highlights the need for higher-quality evidence. For any ICH in the reference cohort, an additional downgrade was applied for serious risk of bias due to non-random imaging attrition, maintaining a Very Low rating. Full downgrading rationales are detailed in Supplementary Table S14.

## 5 Conclusion

Based on very low-certainty evidence (GRADE assessment: starting level “Low” for observational single-arm design, downgraded for indirectness and imprecision), this updated meta-analysis suggests that intravenous thrombolysis following idarucizumab reversal in dabigatran-associated acute ischemic stroke yields pooled symptomatic intracranial hemorrhage and 90-day favorable functional outcome proportions of approximately 5.4 and 66.6%, respectively. However, given the inherent methodological limitations and high uncertainty surrounding the true effect size, these observational findings cannot currently inform routine clinical practice. Instead, they serve primarily as hypothesis-generating benchmarks and provide preliminary *a priori* parameters for sample size estimation and trial design in future confirmatory randomised controlled trials (27). In summary, current evidence suggests that idarucizumab-facilitated intravenous thrombolysis is a feasible strategy with a manageable safety profile in dabigatran-associated AIS. However, given the very low certainty of evidence, these findings should be interpreted as hypothesis-generating. Future prospective registries and randomised controlled trials are warranted to provide higher-level evidence and further define the optimal treatment window and patient selection criteria.

## Data Availability

The original contributions presented in the study are included in the article/Supplementary material, further inquiries can be directed to the corresponding authors.
