# Supplementary material for: Safety and efficacy of idarucizumab-assisted thrombolysis in dabigatran-related acute ischemic stroke: a systematic review and single-arm meta-analysis
Source: Front Neurol. 2026 Aug 20;17:1884021. doi: 10.3389/fneur.2026.1884021 (PMC13537946; doi:10.3389/fneur.2026.1884021)
Supplement: Supplementary file 1 [file Table_1.docx]

**Supplementary Table S1.** PRISMA 2020 Checklist

| Section and Topic | Item # | Checklist item | Location (section/heading) |
| --- | --- | --- | --- |
| **TITLE** |  |  |  |
| Title | 1 | Identify the report as a systematic review. | Title page |
| **ABSTRACT** |  |  |  |
| Abstract | 2 | See the PRISMA 2020 for Abstracts checklist. | Abstract (structured) |
| **INTRODUCTION** |  |  |  |
| Rationale | 3 | Describe the rationale for the review in the context of existing knowledge. | Introduction (1.1–1.2) |
| Objectives | 4 | Provide an explicit statement of the objective(s) or question(s). | Introduction (1.3) |
| **METHODS** |  |  |  |
| Eligibility criteria | 5 | Specify the inclusion and exclusion criteria. | Methods 2.3 |
| Information sources | 6 | Specify all databases, registers, etc. searched. | Methods 2.2 |
| Search strategy | 7 | Present the full search strategies. | Methods 2.2 + Supplementary Table S3 |
| Selection process | 8 | Specify methods used to decide whether a study met inclusion criteria. | Methods 2.3 |
| Data collection process | 9 | Specify methods used to collect data from reports. | Methods 2.4 |
| Data items | 10 | List and define all outcomes. | Methods 2.4 |
| Study risk of bias assessment | 11 | Specify methods used to assess risk of bias. | Methods 2.5 |
| Effect measures | 12 | Specify for each outcome the effect measure(s). | Methods 2.6 |
| Synthesis methods | 13a | Describe processes to decide which studies were eligible for each synthesis. | Methods 2.6 |
|  | 13b | Describe methods to prepare data for synthesis. | Methods 2.6 |
|  | 13c | Describe methods used to tabulate or visually display results. | Methods 2.6 |
|  | 13d | Describe methods used to synthesize results (e.g., random-effects). | Methods 2.6 |
|  | 13e | Describe methods to explore possible causes of heterogeneity. | Methods 2.6, 3.6 |
|  | 13f | Describe sensitivity analyses. | Methods 2.6, 3.6 |
| Reporting bias assessment | 14 | Describe methods to assess risk of bias due to missing results. | Methods 2.6, 3.7 |
| Certainty assessment | 15 | Describe methods to assess certainty (or confidence) in evidence. | Methods 2.6, 4.7 |
| **RESULTS** |  |  |  |
| Study selection | 16a | Describe results of search and selection process. | Results 3.1 + Figure 1 |
|  | 16b | Cite studies that appeared to meet inclusion but were excluded. | Results 3.1 |
| Study characteristics | 17 | Cite each included study and present its characteristics. | Results 3.2 + Table 1 |
| Risk of bias in studies | 18 | Present assessments of risk of bias for each included study. | Results 3.3 + Figure 2 + Supplementary Table S8 |
| Results of individual studies | 19 | For all outcomes, present summary statistic and effect estimate per study. | Results 3.4 (Forest plots) |
| Results of syntheses | 20a | For each synthesis, present summary estimate and its precision. | Results 3.4 (5.4%, 66.6%; 95% CIs) |
|  | 20b | Present results of investigation of heterogeneity. | Results 3.4 (τ², Q test P, prediction intervals) |
|  | 20c | Present results of sensitivity analyses. | Results 3.6 |
| Reporting biases | 21 | Present assessments of risk of bias due to missing results. | Results 3.7 (Doi plots, LFK indices) |
| Certainty of evidence | 22 | Present assessments of certainty in the body of evidence. | Results 4.7 (GRADE) |
| **DISCUSSION** |  |  |  |
| Discussion | 23a | Provide general interpretation of the results. | Discussion 4.1 |
|  | 23b | Discuss limitations of the evidence. | Discussion 4.4 |
|  | 23c | Discuss implications for practice and research. | Discussion 4.3, 4.5 |
| **OTHER INFORMATION** |  |  |  |
| Registration and protocol | 24a | Provide registration information. | Methods 2.1 (PROSPERO CRD420261378203) |
|  | 24b | Indicate where the protocol can be accessed. | Methods 2.1 (PROSPERO) |
|  | 24c | Describe and explain any amendments to registration information. | Methods 2.1 (major revision on 2026-05-12) |
| Support | 25 | Describe sources of financial or non-financial support. | End of manuscript (Funding: None) |
| Competing interests | 26 | Declare any competing interests of review authors. | End of manuscript (Conflict of Interest: None) |
| Availability of data | 27 | Report whether study data are available and from where. | End of manuscript (Data Availability Statement) |

**Supplementary Figure S2.** Screenshot of the PROSPERO record dashboard (CRD420261378203). The dashboard shows the record status after a major revision submitted on 12 May 2026 (version 2.0). The revision narrowed the scope from all DOACs to dabigatran plus idarucizumab and added the Meinel 2023 cohort as an external historical reference. The revision was made after data extraction and quality appraisal but prior to final data synthesis, based on objective evidence availability. The revision history is publicly accessible via PROSPERO.

**Supplementary Table S3.** Complete Search Strategies for All Databases and Registers

| Database | Platform / Interface | Search Date | Full Search Strategy | Filters / Limits |
| --- | --- | --- | --- | --- |
| PubMed | PubMed (NIH) | 2026-04-29 | ("Direct Oral Anticoagulants"[Mesh] OR dabigatran[tiab] OR rivaroxaban[tiab] OR apixaban[tiab] OR edoxaban[tiab]) AND ("Ischemic Stroke"[Mesh] OR "Ischemic Stroke"[tiab]) AND ("Thrombolytic Therapy"[Mesh] OR "Alteplase"[Mesh] OR "Tenecteplase"[Mesh] OR IVT[tiab]) AND ("Antidotes"[Mesh] OR "Idarucizumab"[Mesh] OR "Andexanet Alfa"[Mesh] OR idarucizumab[tiab]) | Language: English/Chinese; Species: Humans; Date: 2010–2026 |
| Embase | Elsevier | 2026-04-29 | ('direct oral anticoagulant'/exp OR dabigatran:ti,ab OR rivaroxaban:ti,ab OR apixaban:ti,ab OR edoxaban:ti,ab) AND ('ischemic stroke'/exp OR 'brain infarction'/exp OR 'ischemic stroke':ti,ab) AND ('thrombolysis'/exp OR 'thrombolytic therapy'/exp OR IVT:ti,ab OR alteplase:ti,ab OR tenecteplase:ti,ab) AND ('antidote'/exp OR idarucizumab:ti,ab OR 'andexanet alfa':ti,ab) | Language: English/Chinese; Humans; Date: 2010–2026 |
| Web of Science | Clarivate Analytics | 2026-04-29 | TS=("direct oral anticoagulant" OR dabigatran OR rivaroxaban OR apixaban OR edoxaban) AND TS=("ischemic stroke" OR "brain infarction") AND TS=(thrombolysis OR IVT OR alteplase OR tenecteplase) AND TS=(antidote OR idarucizumab OR "andexanet alfa") | Language: English/Chinese; Document Types: Article, Review; Date: 2010–2026 |
| Cochrane Library | Wiley | 2026-04-29 | (#1 MeSH descriptor: [Direct Oral Anticoagulants] explode all trees) OR (dabigatran OR rivaroxaban OR apixaban OR edoxaban):ti,ab,kw AND (#2 MeSH descriptor: [Ischemic Stroke] explode all trees) OR ("ischemic stroke"):ti,ab,kw AND (#3 MeSH descriptor: [Thrombolytic Therapy] explode all trees) OR (thrombolysis OR IVT OR alteplase OR tenecteplase):ti,ab,kw AND (#4 MeSH descriptor: [Antidotes] explode all trees) OR (idarucizumab OR "andexanet alfa"):ti,ab,kw | Language: English/Chinese; Trials and Reviews; Date: 2010–2026 |
| CNKI | CNKI (China) | 2026-04-29 | (SU='达比加群' OR SU='直接口服抗凝药' OR SU='利伐沙班' OR SU='阿哌沙班' OR SU='依度沙班') AND (SU='急性缺血性卒中' OR SU='脑梗死') AND (SU='静脉溶栓' OR SU='阿替普酶' OR SU='替奈普酶') AND (SU='逆转' OR SU='依达赛珠单抗' OR SU='拮抗剂') | Language: Chinese; Source: Core Journals/Thesis; Date: 2010–2026 |
| Wanfang Data | Wanfang | 2026-04-29 | (题名或关键词:达比加群 OR 直接口服抗凝药 OR 利伐沙班 OR 阿哌沙班 OR 依度沙班) AND (题名或关键词:急性缺血性卒中 OR 脑梗死) AND (题名或关键词:静脉溶栓 OR 阿替普酶 OR 替奈普酶) AND (题名或关键词:逆转 OR 依达赛珠单抗) | Language: Chinese; Date: 2010–2026 |
| VIP (CQVIP) | Chongqing VIP | 2026-04-29 | (M=达比加群 OR M=直接口服抗凝药 OR M=利伐沙班 OR M=阿哌沙班 OR M=依度沙班) AND (M=急性缺血性卒中 OR M=脑梗死) AND (M=静脉溶栓 OR M=阿替普酶 OR M=替奈普酶) AND (M=逆转 OR M=依达赛珠单抗) | Language: Chinese; Date: 2010–2026 |
| SinoMed | SinoMed (CBM) | 2026-04-29 | ("达比加群"[智能题名] OR "直接口服抗凝药"[智能题名] OR "利伐沙班"[智能题名] OR "阿哌沙班"[智能题名] OR "依度沙班"[智能题名]) AND ("急性缺血性卒中"[智能题名] OR "脑梗死"[智能题名]) AND ("静脉溶栓"[智能题名] OR "阿替普酶"[智能题名] OR "替奈普酶"[智能题名]) AND ("逆转"[智能题名] OR "依达赛珠单抗"[智能题名]) | Language: Chinese; Date: 2010–2026 |
| WHO ICTRP | WHO | 2026-04-29 | Condition: "ischemic stroke" OR "cerebral infarction" OR "brain infarction" AND Intervention: "dabigatran" OR "rivaroxaban" OR "apixaban" OR "edoxaban" OR "idarucizumab" OR "andexanet alfa" OR "reversal agent" | Date: 2010–2026 |
| [ClinicalTrials.gov](https://clinicaltrials.gov/) | NIH | 2026-05-04 | Condition: Ischemic Stroke OR Cerebral Infarction AND Intervention: dabigatran OR rivaroxaban OR apixaban OR edoxaban OR idarucizumab OR andexanet alfa OR reversal | All studies; Date registered: 2010–2026 |

**Supplementary Table S4.** Comparison of key baseline characteristics between the single‑arm meta‑analysis cohort and the Meinel 2023 external reference cohort (DOAC without reversal).

| Characteristic | Single‑arm meta‑analysis cohort  (11 studies, *n*=226) | Meinel 2023 reference cohort (*n*=580) |
| --- | --- | --- |
| Age, median (IQR) | 74–77 (range) | 79.5 (72–84) |
| Baseline NIHSS, median (IQR) | 7–16 (range) | 12 (6–18) |
| Onset‑to‑thrombolysis time, min (IQR) | Varied (not uniformly reported) | 145 (97–190) |
| Mechanical thrombectomy, % | 0–37.6% (varied) | 37.6% |
| DOAC type | Dabigatran 100% | Mixed (rivaroxaban, apixaban, edoxaban, dabigatran) |
| sICH definition | ECASS‑II/SITS‑MOST/unclear | ECASS‑III |
| Outcome completeness | sICH 100%; 90‑day mRS 55%  (6/11 studies) | sICH 100%; 90‑day mRS 100% |

***Footnotes***: Direct statistical comparison was not performed due to inherent differences in study design and lack of individual patient data. The Meinel 2023 cohort is the only available source reporting both sICH and 90‑day mRS in DOAC‑treated patients without reversal.

**Supplementary Material S5.** R Analysis Code

This R script was used to perform the single‑arm meta‑analyses, generate forest plots, and conduct sensitivity/subgroup analyses. It corresponds to the statistical methods described in Section 2.6 of the main manuscript. The script was executed in R version 4.5.3 with the 'meta' package (version ≥ 6.5.0).

**--------------------------------------------------------------------------------**

**Code:**

**--------------------------------------------------------------------------------**

```
#=====================================================================
# Supplementary Material S5: R script for single-arm meta-analysis and descriptive comparison
# Authors: [Your Names]
# Date: 2026-05-13
# R version: 4.5.3
# Required packages: meta (>= 6.5.0)
#=====================================================================
# 1. Load packages ----------------------------------------------------
if (!require(meta)) install.packages("meta")
library(meta)
# 2. Input data --------------------------------------------------------
studies <- c("Wlodarczyk 2023", "Kikule 2022", "Kermer 2020", "Beharry 2020",
             "Fang 2018", "Sanak 2018", "Pikija 2017", "Tse 2017",
             "Frol 2021", "Theodorou 2024", "Romoli 2023")
n_total <- c(19, 9, 80, 13, 10, 13, 5, 6, 22, 10, 39)
# sICH events
sich_events <- c(2, 1, 0, 0, 1, 1, 0, 1, 1, 1, 4)

# Any ICH events
anyich_events <- c(3, 1, 0, 2, 3, 2, 0, 1, 4, 2, 9)
# In-hospital mortality (Kikule, Kermer, Fang, Pikija)
inhosp_studies <- c("Kikule 2022", "Kermer 2020", "Fang 2018", "Pikija 2017")
inhosp_n <- c(9, 80, 10, 5)
inhosp_events <- c(1, 3, 1, 0)
# 90-day mortality (Beharry, Sanak, Frol, Romoli, Tse)
day90_studies <- c("Beharry 2020", "Sanak 2018", "Frol 2021", "Romoli 2023", "Tse 2017")
day90_n <- c(13, 13, 22, 39, 6)
day90_events <- c(2, 3, 3, 7, 1)
# 90-day good functional outcome (mRS 0-2)
good_studies <- c("Beharry 2020", "Sanak 2018", "Tse 2017", "Frol 2021",
                  "Theodorou 2024", "Romoli 2023")
good_n <- c(13, 13, 6, 22, 10, 39)
good_events <- c(8, 10, 2, 19, 5, 25)
# 3. Single-arm meta-analysis (GLMM) ------------------------------------
m_sich <- metaprop(sich_events, n_total, studlab = studies,
                   method = "GLMM", sm = "PLOGIT")
m_anyich <- metaprop(anyich_events, n_total, studlab = studies,
                     method = "GLMM", sm = "PLOGIT")
m_inhosp <- metaprop(inhosp_events, inhosp_n, studlab = inhosp_studies,
                     method = "GLMM", sm = "PLOGIT")
m_day90 <- metaprop(day90_events, day90_n, studlab = day90_studies,
                    method = "GLMM", sm = "PLOGIT")
m_good <- metaprop(good_events, good_n, studlab = good_studies,
                   method = "GLMM", sm = "PLOGIT")
# 4. Print summary results----------------------------------------------
cat("========== sICH ==========\n")
print(summary(m_sich), digits = 3)
cat("\n========== Any ICH ==========\n")
print(summary(m_anyich), digits = 3)
cat("\n========== In-hospital mortality ==========\n")
print(summary(m_inhosp), digits = 3)
cat("\n========== 90-day mortality ==========\n")
print(summary(m_day90), digits = 3)
cat("\n========== 90-day good functional outcome ==========\n")
print(summary(m_good), digits = 3)
# 5. Forest plots (saved to D:/) -----------------------------------------
pdf("D:/Figure3_sICH.pdf", width = 8, height = 6)
forest(m_sich, leftcols = c("studlab", "event", "n"),
       leftlabs = c("Study", "Events", "Total"),
       rightcols = c("effect", "ci"),
       rightlabs = c("Proportion", "95% CI"),
       xlab = "Proportion of sICH", main = "sICH")
dev.off()
pdf("D:/Figure4_goodOutcome.pdf", width = 8, height = 5)
forest(m_good, leftcols = c("studlab", "event", "n"),
       leftlabs = c("Study", "Events", "Total"),
       rightcols = c("effect", "ci"),
       rightlabs = c("Proportion", "95% CI"),
       xlab = "Proportion of good outcome", main = "90-day mRS 0-2")
dev.off()
pdf("D:/Figure5_anyICH.pdf", width = 8, height = 6)
forest(m_anyich, leftcols = c("studlab", "event", "n"),
       leftlabs = c("Study", "Events", "Total"),
       rightcols = c("effect", "ci"),
       rightlabs = c("Proportion", "95% CI"),
       xlab = "Proportion of any ICH", main = "Any ICH")
dev.off()
# 6. Funnel plots and Egger's test ---------------------------------------
pdf("D:/Figure6_funnel.pdf", width = 10, height = 5)
par(mfrow = c(1,2))
funnel(m_sich, xlab = "Proportion of sICH", main = "A) sICH", studlab = FALSE)
egger_sich <- metabias(m_sich, method = "Egger")
text(0.1, 1.2, paste("Egger's test P =", round(egger_sich$p.value, 4)), cex = 0.8)
funnel(m_good, xlab = "Proportion of good outcome", main = "B) Good outcome", studlab = FALSE)
text(0.45, 1.2, "Egger's test not performed (k=6)", cex = 0.8)
dev.off()
# 7. Sensitivity analysis: leave-one-out for sICH ------------------------
m_sich_inf <- metainf(m_sich, pooled = "random")
pdf("D:/Supplementary_Figure_S3.pdf", width = 8, height = 6)
forest(m_sich_inf, xlab = "Proportion of sICH (leave-one-out)",
       main = "Leave-one-out sensitivity analysis for sICH")
dev.off()
# 8. Subgroup analysis by thrombolytic agent (requires drug variable) ------
drug <- c(rep("Alteplase", 10), "Tenecteplase")
m_sub <- metaprop(sich_events, n_total, studlab = studies, subgroup = drug,
                  method = "GLMM", sm = "PLOGIT")
pdf("D:/Supplementary_Figure_S2.pdf", width = 9, height = 6)
forest(m_sub, leftcols = c("studlab", "event", "n"),
       leftlabs = c("Study", "Events", "Total"),
       rightcols = c("effect", "ci"),
       rightlabs = c("Proportion", "95% CI"),
       xlab = "Proportion of sICH",
       main = "Subgroup analysis by thrombolytic agent")
dev.off()
# 9. Session information -----------------------------------------------
sessionInfo()
```

**Supplementary Table S6.** Raw data extraction matrix for included single‑arm studies and external reference cohort

| Study ID | First author | Year | Country | Study design | Analysis intent | Group | Total *N* | sICH (*n*/*N*) | Any ICH *(n*/*N*) | Mortality (*n*/*N*) | Good outcome (*n*/*N*) | Outcome timepoint |
| --- | --- | --- | --- | --- | --- | --- | --- | --- | --- | --- | --- | --- |
| 1 | Wlodarczyk | 2023 | Poland | Retrospective cohort | Pooled_Meta | Idarucizumab + Dabigatran | 19 | 2/19 | 3/19 | 1/19 | 13/19 | Discharge  (mRS 0‑2) |
| 2 | Kikule | 2022 | Latvia | Retrospective cohort | Pooled_Meta | Idarucizumab + Dabigatran | 9 | 1/9 | 1/9 | 1/9 | 4/9 | Discharge  (mRS 0‑2) |
| 3 | Kermer | 2020 | Germany | Retrospective cohort | Pooled_Meta | Idarucizumab + Dabigatran | 80 | 0/80 | 0/80 | 3/80 | NR | No 90‑day data |
| 4 | Beharry | 2020 | Australia/NZ | Retrospective cohort | Pooled_Meta | Idarucizumab + Dabigatran | 13 | 0/13 | 2/13 | 2/13 | 8/13 | 90‑day (mRS 0‑2) |
| 5 | Fang | 2018 | Taiwan (China) | Retrospective cohort | Pooled_Meta | Idarucizumab + Dabigatran | 10 | 1/10 | 3/10 | 1/10 | NR | No 90‑day data |
| 6 | Sanak | 2018 | Czech Republic | Retrospective cohort | Pooled_Meta | Idarucizumab + Dabigatran | 13 | 1/13 | 2/13 | 3/13 | 10/13 | 90‑day (mRS 0‑2) |
| 7 | Pikija | 2017 | Austria | Retrospective cohort | Pooled_Meta | Idarucizumab + Dabigatran | 5 | 0/5 | 0/5 | 0/5 | NR | No 90‑day data |
| 8 | Tse | 2017 | New Zealand | Retrospective cohort | Pooled_Meta | Idarucizumab + Dabigatran | 6 | 1/6 | 1/6 | 1/6 | 2/6 | 90‑day (mRS 0‑2) |
| 9 | Frol | 2021 | Slovenia | Prospective cohort | Pooled_Meta | Idarucizumab + Dabigatran | 22 | 1/22 | 4/22 | 3/22 | 19/22 | 90‑day (mRS 0‑2) |
| 10 | Theodorou | 2024 | Greece | Retrospective cohort | Pooled_Meta | Idarucizumab + Dabigatran | 10 | 1/10 | 2/10 | 2/10 | 5/10 | 90‑day (mRS 0‑2) |
| 11 | Romoli | 2023 | Italy | Retrospective cohort | Pooled_Meta | Idarucizumab + Dabigatran | 39 | 4/39 | 9/39 | 7/39 | 25/39 | 90‑day (mRS 0‑2) |
| 12 | Meinel_Rev | 2023 | International (64 centers) | Retrospective cohort | Descriptive Only | Idarucizumab + Dabigatran | 252 | 3/252 | 16/205* | NR | 136/252 | 90‑day (mRS 0‑2) |
| 13 | Meinel_Rev | 2023 | International (64 centers) | Retrospective cohort | Descriptive Only | DOAC without reversal | 580 | 18/580 | 125/580 | NR | 246/580 | 90‑day (mRS 0‑2) |

**Supplementary Figure S7.** Model diagnostic output for single‑arm meta‑analyses. The figure shows the random‑effects GLMM results for sICH, any ICH, in‑hospital mortality, and 90‑day mortality, as well as the subgroup analysis by thrombolytic agent. Heterogeneity was assessed using τ² and the Q test (no I² reported). For detailed numerical results see Section 3.4. Output generated with R meta package (version 4.5.3).

**Supplementary Table S8.** Sensitivity analysis for sICH definition heterogeneity.

| Analysis set | Studies included | Sample size | sICH rate  (95% *CI*) | Difference from main analysis |
| --- | --- | --- | --- | --- |
| Main analysis (all 11 studies) | 11 | 226 | 5.4% (2.6%–10.6%) | Reference |
| Excluding studies without ECASS‑II/SITS‑MOST (Kermer 2020, Pikija 2017) | 9 | 210 | 5.1% (2.4%–10.4%) | −0.3% |
| Further excluding Kermer 2020 (largest non‑standard study) | 8 | 130 | 6.2% (2.9%–12.8%) | +0.8% |

***Footnote***: Kermer 2020 and Pikija 2017 did not provide predefined sICH criteria. The remaining 9 studies explicitly used ECASS‑II or SITS‑MOST definitions.

**Supplementary Table S9.** JBI Critical Appraisal Checklist for Case Series — summary of included studies.

| Study | Clear aims | Consecutive | Participants described | Demographics | Clinical features | Outcomes clear | Outcome data | Statistical analysis | Adverse events | Long-term outcomes | Overall |
| --- | --- | --- | --- | --- | --- | --- | --- | --- | --- | --- | --- |
| Wlodarczyk 2023 | High | Low | Low | High | High | Low | Low | High | Low | Low | Moderate |
| Kikule 2022 | High | High | Low | High | High | Low | Low | High | Low | Low | Moderate |
| Kermer 2020 | High | Low | Low | High | High | Low | Low | High | Low | Low | Moderate |
| Beharry 2020 | Low | Low | Low | Low | Low | Low | Low | Low | Low | Low | Low |
| Fang 2018 | High | Low | Low | High | High | Low | Low | High | Low | Low | Moderate |
| Sanak 2018 | Low | Low | Low | Low | Low | Low | Low | Low | Low | Low | Low |
| Pikija 2017 | High | Low | Low | High | High | Low | Low | High | Low | Low | Moderate |
| Tse 2017 | High | Low | Low | High | High | Low | Low | Low | Low | Low | Moderate |
| Frol 2021 | Low | Low | Low | Low | Low | Low | Low | Low | Low | Low | Low |
| Theodorou 2024 | Low | Low | Low | Low | Low | Low | Low | Low | Low | Low | Low |
| Romoli 2023 | Low | Low | Low | Low | Low | Low | Low | Low | Low | Low | Low |

***Footnotes***:Clear aims corresponds to D1 (clear inclusion criteria) in the JBI checklist. Consecutive enrollment corresponds to D4 (consecutive recruitment of participants). Participants described corresponds to D3 (valid identification of the condition). Demographics corresponds to D6 (demographic information clearly reported). Clinical features corresponds to D7 (clinical information clearly reported). Outcomes clear and Outcome data collectively correspond to D8 (outcomes or follow‑up results clearly reported). Statistical analysis corresponds to D10 (appropriate statistical analysis). Adverse events is not a core JBI item; it reflects reporting of safety outcomes. Long‑term outcomes is an extension of D8, indicating follow‑up beyond discharge. Overall risk assessment (Low/Moderate) reflects the authors’ judgement based on the pre‑dominant ratings.

**Supplementary Figure S10.** Forest plot of the pooled proportions for in‑hospital and 90‑day mortality. (A) In‑hospital mortality (4 studies). Pooled proportion from random‑effects GLMM: 4.8% (95% CI 2.0%–11.0%). (B) 90‑day mortality (5 studies). Pooled proportion: 17.2% (95% CI 10.8%–26.3%). Heterogeneity was assessed using *τ²* and the Q test. For details see Section 3.4.4.

****Supplementary Table S11.** Descriptive comparative analysis of outcomes between the idarucizumab reversal group and the DOAC group without reversal (Meinel 2023).**

| **Outcome** | **Reversal group**  **(*n*=252)** | **DOAC group without reversal**  **(*n*=580)** |
| --- | --- | --- |
| Symptomatic intracranial hemorrhage (sICH) | 3/252 (1.2%) | 18/580 (3.1%) |
| Good functional outcome at 90 days  (mRS 0–2) | 136/252 (54.0%) | 246/580 (42.4%) |
| Any intracranial hemorrhage | 16/205 (7.8%)* | 125/580 (21.6%) |

***Footnote***: * In the reversal group, the percentage of any intracranial hemorrhage was based on 205 patients who had follow‑up imaging (81.3% of the group), as reported in the original article (7.8%). In the DOAC group without reversal, the percentage was based on all 580 patients. Data are presented descriptively only; no statistical pooling or comparison was performed. The 90‑day mortality was not reported by Meinel 2023 for these subgroups.

**Supplementary Figure S12.** Subgroup analysis by thrombolytic agent (alteplase vs. tenecteplase) for sICH. The analysis used a random‑effects GLMM with a logit link. For the 10 studies using alteplase, the pooled sICH rate was 5.0% (95% CI 2.4%–10.2%). For the single study using tenecteplase (Beharry 2020), the rate was 0.0% (95% CI 0.0%–24.3%). Test for subgroup differences (random effects): *χ²* = 1.61, *df* = 1, *p* = 0.2050. Heterogeneity was quantified using τ². Data are presented as proportions; percentages in the main text are shown with one decimal place for consistency with the primary analysis. See Section 3.6 for details.

**Supplementary Figure S13.** Leave‑one‑out sensitivity analysis for sICH. The forest plot shows the pooled sICH proportion after sequentially omitting each study, estimated using a random‑effects GLMM. The diamond at the bottom represents the overall pooled estimate (5.0%, 95% CI 3.0%–11.0%). No single study exerted a disproportionate influence on the pooled estimate. Data are presented as proportions; percentages in the main text are shown with one decimal place for consistency with the primary analysis. See Section 3.6 for details.

**Supplementary Table S14. GRADE Summary of Findings (SoF) Table for outcomes (sICH, 90‑day good functional outcome, and any intracranial hemorrhage in the external reference cohort).**

**Note**: GRADE certainty assessment applies solely to the idarucizumab + IVT intervention group (single‑arm meta‑analysis). The external reference cohort (Meinel 2023) is presented descriptively per PRISMA‑SWiM guidance and is **not** subject to formal comparative GRADE grading. The “Very low” rating for this cohort reflects the low quality of the source observational study, not a comparative inference.

| **Outcome** | **№ of studies**  **(patients)** | **Estimated event rate per 1000 patients**  **(95% CI)** | **Certainty assessment** | **Overall certainty of evidence** | **Explanations** |
| --- | --- | --- | --- | --- | --- |
| **sICH** | 11 (226) | 54 per 1000 (26 to 106) | No serious inconsistency; no serious risk of bias*; serious indirectness†; serious imprecision‡ | **⊕◯◯◯ Very low** | *GLMM handled zero events; JBI assessment performed. †Single‑arm with descriptive external reference cannot answer comparative question. ‡Wide CI, limited sample size (226), low events (12). |
| **90‑day good functional outcome (mRS 0‑2)** | 6 (103) | 666 per 1000 (542 to 771) | No serious risk of bias; **no statistically significant heterogeneity (τ²=0.116, Q test P=0.12)**; serious indirectness†; serious imprecision‡ | **⊕◯◯◯ Very low** | †Same as above. ‡Wide CI and limited sample size (103). |
| **Any intracranial hemorrhage (external reference cohort)** | 1 (252*) | 78 per 1000 (N/A) | Serious risk of bias§; serious indirectness; serious imprecision; single study only | **⊕◯◯◯ Very low** | §18.4% non‑random missing imaging data (47 of 252 patients) introduces attrition bias. Only 205 patients (81.3%) had follow‑up imaging; the event rate (7.8%) is based on those 205. |

**Footnotes: Starting level of evidence** was set to “Low” (observational single‑arm design). **Downgrading rule**: each limitation reduces certainty by one level. **Interpretation of Very low certainty**: The true effect is likely to be substantially different from the estimate, and further research is very likely to change the confidence in the estimate. **Absolute effect calculation**: Because this is a single‑arm meta‑analysis without a concurrent control group, the “estimated event rate per 1000 patients” is presented instead of a comparative absolute effect. * The total group size is 252, but only 205 patients (81.3%) had follow‑up imaging for any intracranial hemorrhage; the event rate is therefore presented as 16/205 (7.8%). The 47 missing imaging studies represent non‑random missing data (reason not reported in the original article). This bias does not affect the pooled sICH estimate from the single‑arm meta‑analysis. **Abbreviations**: CI, confidence interval; GLMM, generalized linear mixed model; JBI, Joanna Briggs Institute; mRS, modified Rankin Scale; N/A, not applicable; sICH, symptomatic intracranial hemorrhage; τ², between‑study variance.
